# High Compliance with Newborn Community-to-Facility Referral in Eastern Uganda:.An Opportunity to Improve Newborn Survival

**DOI:** 10.1371/journal.pone.0081610

**Published:** 2013-11-29

**Authors:** Christine Kayemba Nalwadda, Peter Waiswa, Juliet Kiguli, Gertrude Namazzi, Sarah Namutamba, Göran Tomson, Stefan Peterson, David Guwatudde

**Affiliations:** 1 School of Public Health, College of Health Sciences, Makerere University, Kampala, Uganda; 2 Uganda Newborn Survival Study, Iganga- Mayuge Demographic Surveillance Site, Kampala, Uganda; 3 Health System Policy, Department of Public Health Sciences, Karolinska Institutet, Stockholm, Sweden; 4 Medical Management Center (MMC) Karolinska Institutet, Stockholm , Sweden; 5 International Maternal and Child Health Unit, Department of Women and Children Health, Uppsala University, Uppsala, Sweden; The Ohio State University, United States of America

## Abstract

**Background:**

Seventy-five percent of newborn deaths happen in the first-week of life, with the highest risk of death in the first 24-hours after birth.WHO and UNICEF recommend home-visits for babies in the first-week of life to assess for danger-signs and counsel caretakers for immediate referral of sick newborns. We assessed timely compliance with newborn referrals made by community-health workers (CHWs), and its determinants in Iganga and Mayuge Districts in rural eastern Uganda.

**Methods:**

A historical cohort study design was used to retrospectively follow up newborns referred to health facilities between September 2009 and August 2011. Timely compliance was defined as caretakers of newborns complying with CHWs’ referral advice within 24-hours.

**Results:**

A total of 724 newborns were referred by CHWs of whom 700 were successfully traced. Of the 700 newborns, 373 (53%) were referred for immunization and postnatal-care, and 327 (47%) because of a danger-sign. Overall, 439 (63%) complied, and of the 327 sick newborns, 243 (74%) caretakers complied with the referrals. Predictors of referral compliance were; the newborn being sick at the time of referral- Adjusted Odds Ratio (AOR) = 2.3, and 95% Confidence-Interval (CI) of [1.6 - 3.5]), the CHW making a reminder visit to the referred newborn shortly after referral (AOR =1.7; 95% CI: [1.2 -2.7]); and age of mother (25-29) and (30-34) years, (AOR =0.4; 95% CI: [0.2 - 0.8]) and (AOR = 0.4; 95% CI: [0.2 - 0.8]) respectively.

**Conclusion:**

Caretakers’ newborn referral compliance was high in this setting. The newborn being sick, being born to a younger mother and a reminder visit by the CHW to a referred newborn were predictors of newborn referral compliance. Integration of CHWs into maternal and newborn care programs has the potential to increase care seeking for newborns, which may contribute to reduction of newborn mortality.

## Introduction

The prevailing high levels of newborn deaths are a major barrier to the achievement of the fourth millennium development goal (MDG-4) particularly in Sub Saharan Africa (SSA) where the risk of death during the neonatal period is highest [1]. MDG-4 aims at reducing child mortality levels of 1990, by two thirds by 2015. Globally, 43% of child deaths are of newborns [1], with 99% of them occurring in low income countries (LICs) and most die at home [2,3]. In Uganda, the Neonatal Mortality Rate (NMR) is 27.0 deaths per 1000 live births [4], compared to 35.9 in Africa [2].

Globally, the leading causes of newborn deaths are complications from preterm births (29%), asphyxia (23%) and infections due to sepsis and pneumonia (25%) [5]. Other causes include delays to get health care for sick newborns. In Uganda, Waiswa et al., demonstrated that newborn deaths are mainly due to delays in; recognizing the illness and making decision to seek care (50%), reaching a health facility (20%), and receiving quality care at the health service point (30%) [6].

Newborn referral from communities to health facilities is a strategy that has been shown to improve newborn survival in south Asia [7,8]. Community newborn referrals can be initiated by community health workers (CHWs) [9,10,11,12], traditional birth attendants (TBAs) [13], or self-referrals [11]. However, for newborns to benefit from the referrals, their caretakers (mothers or guardians) should comply with referral advice in a timely manner to increase chances of survival. 

 In Uganda, the level of and the determinants of compliance with newborn referral are not known. Existing data focus on older children aged 1- 59 months. For instance, under the Integrated Management of Child Illnesses strategy, compliance with referral from health centres to district hospitals among under-fives and children aged one week to 2 months was 28% and 21%, respectively. In another study focusing on home-based management of fever program, compliance with community drug distributors’ referral advice from community to health facilities, was 93% among under-five children [14,15]. In south Asia, compliance to newborn referrals facilitated by CHWs was reported to range between 30 - 54% [16,17], suggesting existence of substantial barriers to newborn care seeking. 

Therefore a critical knowledge gap exists about newborn referral compliance and its determinants in SSA. Hence, this study evaluated timely compliance with newborn referrals initiated by CHWs, and its determinants in eastern Uganda.

## Materials and Methods

### Study setting

This study was conducted between June and July 2012 at the Iganga-Mayuge Health Demographic Surveillance Site (HDSS) [18] located 78 kilometres (km) east of Kampala, the capital city of Uganda. The HDSS has about 70,000 people, living in 65 villages, of which 13 are peri-urban forming Iganga town council and the rest are rural. The majority of the population practice subsistence farming for a living. Over half of the population (56%) are below 18 years of age. Only 57% of all child births occur at health facilities [4]. This study was conducted as an add-on to the Uganda Newborn Survival Study (UNEST) within the HDSS, details of which are described elsewhere [19]. The UNEST provided a good opportunity to evaluate compliance with newborn referrals initiated by CHWs.

### Study design and population

A historical cohort study design was used. Registers maintained by the CHWs under the UNEST were reviewed to identify newborns that were referred to health facilities between September 2009 and August 2011. All newborns referred during this period were eligible for inclusion in this study. Location details recorded on the referral forms were used to trace the caretakers of identified newborns in the community for interview.

During the conduct of the UNEST, a CHW referred either a sick newborn for appropriate care, or a newborn delivered outside a health facility for immunisation and postnatal care. Since the first day of life bears the highest risk of death for newborns, CHWs were trained to counsel all caretakers with referred newborns to comply with the referral immediately. CHWs were also required to make a follow up visit to the referred newborns as a reminder to comply with the referral within 24-hours. A sick newborn was defined as one identified by the CHW to exhibit one or more of the newborn danger signs adopted from the WHO-UNICEF algorithm. The newborn danger signs included; difficulty in breathing, fever, coldness, yellowing of eyes and body, severe skin rash with pus, diarrhoea, vomiting excessive crying, very weak, failure to breastfeed, convulsions and an umbilical cord with pus or redness [20].

The CHWs completed a referral form for each newborn they referred. The form indicated the village, parish and sub county of residence , names of the newborn, mother and head of household, age and dates of birth and referral of the newborn, reason for referral, in addition to the name of the referring CHW.

 A team of the Iganga-Mayuge HDSS research assistants was trained on the objectives and study tools of this study for three days, including one day of pre-testing. During data collection, the completed referral forms served two purposes; i) they provided some data (to minimise recall bias by caretakers) and ii) they acted as guiding tools to trace the households of identified newborns in the community. On successful tracing of the household of the identified newborn, the study objectives were explained to the caretaker of the newborn (identified as the person in the household who provided most of the day-to-day care to the newborn), and written informed consent sought. 

The study questionnaire was then administered to the caretaker. The questionnaire collected data including; i) demographics of the caretaker at the time of referral, ii) date and age of the newborn at referral iii) reason for referral of the newborn (ii and iii were obtained from the referral forms), iv) number of CHW visits to mother during and after pregnancy v) place of delivery of the newborn, vi) clinical characteristics of the newborn at the time of referral, vii) compliance with referral advice given by CHW, viii) reason(s) for non-compliance, ix) follow up visit to referred newborn by CHW, x) place where referral care was sought, xi) distance to the place where referral care was sought, and xii) household ownership of selected assets for classification of social economic status (SES) of the newborn household. The questions were adopted from existing tools used in UNEST.

### Statistical analysis strategy

Double data entry was done using the EPIDATA statistical package. Data were cleaned and exported to STATA statistical package version 10 for analysis. Univariate statistics were used to describe the characteristics of the newborns and their caretakers. Further, proportions of the caretakers who complied with the newborn referral were calculated, using all newborns enrolled in this study as the denominator. Referral compliance in this study was defined as a health facility visit by the newborn caretaker within 24-hours following a CHW’s assessment of a newborn and issuing a referral form. Caretakers who reported to health facilities after 24-hours or did not report at all were considered non-compliant. The 24-hour time frame was selected given that the highest risk of newborn death is within this period of life [21]. Since compliance is also influenced by several factors, a 24-hour period was deemed realistic for the caretakers to respond positively. Other authors have also defined delay in compliance with newborn referral using the same timeline [15,17].

To classify the SES of households, principal component analysis (PCA) was run on 12 household assets evaluated. These assets included; i) source of drinking water mostly used, ii) toilet facility used, iii) type of housing, iv) number of rooms, v) material of floor, roof, and walls of the house, vi) Type of cooking fuel, vii) lighting source, viii) tenure of the house ix) ownership of any of the following: cattle, sheep, goat, pig or chicken, x) ownership of land, xi) number of cooked meals consumed in day, and xii) ownership of any of the following: bicycle, motorcycle, radio, table, bed, mosquito net, CD player, mobile phone, television set or farm implements. The principal component on which most assets loaded (the first principal component) was used to generate an SES score for each newborn’s household. The households were then grouped into five descending SES quintiles, with higher quintile indicating higher SES. This approach has also been used in Demographic and Health Surveys in Uganda [4], and Mayega et al [22].

To identify factors associated with compliance with newborn referral, multi-variable logistic regression analysis was used, with timely newborn referral compliance as the binary outcome. Before conducting multivariable analysis, we investigated existence of multicolinearlity using the correlation coefficient between each pair of the independent variables. If any two variables were found to have a correlation coefficient value greater than 0.5 with a p-value less or equal to 0.05, one of them was excluded in the multivariable analysis logistic regression analysis, retaining the one with a higher p-value in the model. All the remaining independent variables initially included in the logistic regression model. One was removed at a time, starting with the variable with the highest p-value, until a final model was obtained containing only variables with a p-value less or equal to 0.05. The independent factors investigated included; i) demographic and background characteristics of the caretakers, heads of households and newborns ii) place of delivery of referred newborn, iii) reason for newborn referral, iv) SES of the household, v) newborn symptoms present at referral including difficulty in breathing, yellow eyes and body severe skin rash with pus, excessive crying, and newborn being very weak difficulty in breastfeeding; vi) caretaker’s perception of health condition of the newborn, and xiii) CHWs going back to check on referred newborns shortly after referral.

The Hosmer-Lemeshow test was used to assess the goodness of fit of the logistic regression model. The final model had a Hosmer-Lemeshow test p-value of greater than 0.05, which was considered an adequate fit to the data [23]. We report crude odd ratios (COR) and adjusted odd ratios (AOR) with their respective 95% confidence intervals (CI) and p-values as measures of association.

### Ethical statement

The study protocol was approved by the Makerere University, College of Health Sciences, School of Public Health Higher Degrees Research Committee and the National Council of Science and Technology (Ref. SS2660). Written informed consent was sought from all study participants prior to the interviews and confidentiality observed by use of identification numbers for the respondents. Permission was also sought from the Iganga-Mayuge HDSS management. Data from this study is available on request with justification from the corresponding author of this article.

## Results

### Background characteristics of the caretakers and newborns

A total of 724 newborns were referred by the CHW between September 2009 and August 2011; of which 700 were successfully traced and all of their caretakers consented to participate in this study. Caretakers of 24 newborns were reported to have changed residence to unknown addresses. A total of 689 of the 700 respondents (98%) were mothers to the newborns, with a mean age of 27 years (SD 6.4 years). The majority of the caretakers 648 (93%) were currently married. Of the 631 caretakers who reported to have ever attended school, 46% had completed 7 years of formal education. Of the 700 newborns, 51% were males, and 56% were delivered at a health facility. The rest of the characteristics are presented in Table 1.

**Table 1:** Background characteristics of caretakers and newborns.

**Characteristics**	**Category**	**Freq (N=700)**	**Percentage %**
**Caretakers**			
Age of mother of newborn§	<20	53	8.6
	20 - 24	178	28.9
	25 - 29	153	24.9
	30 - 34	141	22.9
	35 - 39	55	8.9
	40+	35	5.7
Marital status of caretaker	Never married	21	3.0
	Currently married	648	92.6
	Widowed	6	0.8
	Divorced /separated	25	3.6
Relationship of caretaker to newborn	Mother	689	98.4
	Other	11	1.6
Ethnicity of caretaker	Musoga	565	80.7
	Others	135	19.3
Caretaker ever attended school	Yes	631	90.1
	No	69	9.9
Years of formal education completed by caretaker	<7(Primary education)	288	45.6
	≥7(Post primary)	343	54.4
**Newborns**			
Sex of newborn	Male	342	48.8
	Female	359	51.2
Age of newborn at referral	≤7 days	607	86.7
	>7 days	93	13.3
Place of birth of newborn	At facility	389	55.7
	Outside facility	311	41.3

^§^ N=615

**Figure 1:**
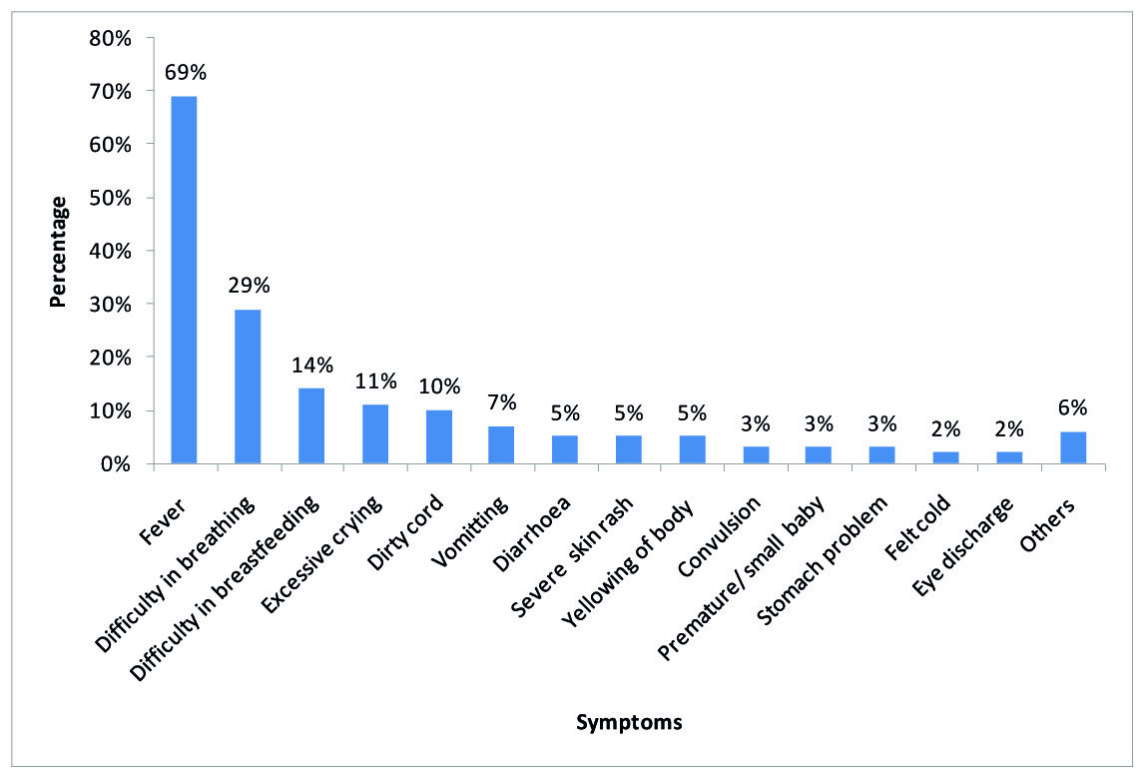
Symptoms reported by caretakers of newborns referred due to sickness.

Of the 700 newborns, 87% were referred during the first week of life at a median age of 3 days (range 2-7). A total of 373 (53%) were referred for immunisation and postnatal care, and the rest referred due to sickness. Among those referred due to sickness, the most common symptoms reported included; fever (69%), difficulty in breathing (29%), difficulty in breastfeeding (14%), excessive crying (11%), and an umbilical cord with pus or bleeding (10%) (Figure 1).

### Newborn Referral Compliance

Of the 700 newborns referred, 640 caretakers (91%) had sought referral care, regardless of the time taken, with a median time of 16 hours (range 3-48). However, 439 (63%) of the caretakers had complied with the referral advice within 24-hours, with a median time of 13 hours (range 2-17). Out of 327 newborns referred due to sickness, 243 (74%) caretakers complied within 24-hours compared to 196 of the 373 (53%) referred for immunisation and postnatal care (p<0.001) (Table 2).

**Table 2:** Compliance rates with newborn referral by selected characteristics of newborns and caretakers.

**Characteristic**	**Group**	**n**	**Complied with 24 hours**	**Did not Comply with 24 hours**	**p-value**
Age of newborn at referral	1 wk	607	374 (61.6)	233 (38.4)	
	> 1 wk	93	65 (69.9)	28 (30.1)	0.125
Reason for referral	Sick	327	243 (74.3)	84 (25.7)	
	Immunisation	373	196 (52.6)	177 (47.5)	<0.001 *
Place of delivery	Facility	389	260 (66.8)	129 (33.2)	
	Outside facility	311	179 (57.6)	132 (42.4)	0.012*
CHW visits in pregnancy	<3 times	591	370 (62.6)	221 (37.4)	
	≥3 times	109	69 (63.3)	40 (36.7)	0.890
CHW visits after delivery	<3 times	648	408 (63.3)	240 (37.0)	
	≥3 times	52	31 (59.6)	21 (40.4)	0.631
Reminder visit by CHW	No	153	78 (51.0)	75 (49.0)	
	Yes	533	353 (66.2)	180 (33.8)	0.001*
Wealth quintiles (Poor to least poor)	1^st^	135	79(58.5)	56 (41.5)	
	2^nd^	135	85(63.0)	50 (37.0)	0.455
	3^rd^	135	90 (66.7)	45 (33.3)	0.167
	4^th^	135	80(59.3)	55 (40.7)	0.902
	5^th^	135	88(65.1)	47 (34.8)	0.260

^*^ Statistically significant <0.05

Among the 261 caretakers who did not comply with the referral advice, 60 (23%) did not seek referral care at all, while the rest 201 (77%) sought care after 24-hours. Of the 60 caretakers who did not seek care at all, 39 (65%) were those whose newborns were referred due to sickness.

### Care for referred newborns

Of the 640 caretakers who sought referral care, 493 (77%) went to public health centres, 115 (18%) to hospital, and 32 (5%) to other facilities. The most common means of transport to seek referral care from the health facilities was “walking,” used by 47% (301/640) of the caretakers. The mean distance from the caretakers’ residence to the health facility where referral care was sought was 2 km (SD1.6). 

Out of the 39 sick newborns whose caretakers did not seek referral care at all, 27 (68%) were treated at home. The caretakers treated these sick newborns either with medicines newly bought 21 (54%), or with old-stock drugs kept at home 6 (15%).

### Reasons for non-compliance

Caretakers who did not comply (261) mentioned several reasons for non –compliance. Fifty (19%) of the caretakers did not comply because “the mother was sick (unwell)”, 45 (17%) caretakers were “waiting for an immunization outreach” services in their area, and 39 (15%) were “waiting for the newborn’s umbilical cord to drop off”. A summary of the rest of the reasons is presented in Table 3.

**Table 3:** Barriers for timely compliance reported by caretakers of referred newborns by reason for referral.

**Reason for non- compliance**	**Sick**	**Immunisation**	**Overall**
	**(n=84)**	(**n=177**)	(**N=261**)
	**Freq (%)**	**Freq (%**)	**Freq (%)**
Mother sick	14 (16.7)	36 (20.3)	50 (19.2)
Waiting for immunisation outreach	2 (2.4 )	43 (24.3)	45 (17.2)
Waiting for umbilical cord to drop off	3 (3.6)	36 (24.3)	39 (14.9)
Lack of Transport	12 (14.3)	10 (5.7)	22 (8.4)
Health workers absent during weekend	5 (6.0)	5 (2.8)	10 (3.8)
CHW advised mother to go after 1 week	1(1.2)	6 (3.4)	7 (2.7)
Bad weather	4 (4.8)	1 (0.6)	5 (1.9)
Mother busy with work at home	2 (2.4)	3 (1.7)	5 (1.9)
Long distance to facility	0 (0.0)	3 (1.7)	3 (1.2)
Had sick person at home	0 (0.0)	3 (1.7)	3 (1.2)
Needed permission from husband	1 (1.2)	2 (1.2)	3 (1.2)
Others	8 (9.5)	8 (4.5)	16 (9.0)

### Factors associated with newborn referral compliance

Age of the mother, reason for referral of newborn and a reminder visit to the referred newborn by CHW within 24-hours were the factors found to be significantly associated with compliance with referral advice given by CHWs.

Mothers aged (25-29 and 30-34) years were both 0.4 times less likely to comply with newborn referral advice compared to younger mothers aged less than 20 years; Adjusted Odd Ratio (AOR) = 0.4; and 95% Confidence Interval (CI) of [0.2 - 0.8]) and (AOR = 0.4; 95% CI: [0.2 - 0.8]) respectively. Newborns referred because they were sick (suffered a danger sign) were 2.3 times more likely to comply with the referral advice compared to those who were referred for immunization and postnatal care; (AOR 2.3; 95% CI: [1.6 - 3.5]). However, no specific danger sign was associated with compliance with referral advice. Caretakers who received a reminder visit by the CHW within 24-hours following initial referral advice were 1.7 times more likely to comply with the referral advice compared to those who did not receive such a visit; (AOR 1.7; 95% CI: [1.2 - 2.7]). The rest of the results are summarized in Table 4.

**Table 4:** Multi- variable regression with timely compliance by selected caretaker and newborn characteristics.

**Characteristic**	**Category**	**n-**	**# Complying within 24 hrs (%)**	**Crude OR [95%CI]**	**Adjusted OR [95% CI]**
Age of mother	<20	53	12 (22.6)	1.0	
	20 - 24	178	58 (32.6)	0.6 [0.3 - 1.2]	0.6 [0.3 - 1.3]
	25 - 29	153	66 (43.1)	0.4 [0.2 - 0.8]	0.4 [0.2 - 0.8]
	30 - 34	141	59 (41.8)	0.4 [0.2 - 0.8]	0.4 [0.2 - 0.8]
	35 - 39	55	20 (36.4)	0.5 [0.2 - 1.2]	0.5 [0.2 - 1.3]
	≥40	35	13 (37.1)	0.5 [0.2 - 1.3]	0.4 [0.1 - 1.2]
Reason for referral of newborn	Immunisation	373	196 (52.6)	1.0	
	Sick	327	243 (74.3)	2.6 [1.9 -3.6]	2.3 [1.6 - 3.5]
Age of newborn at referral	<1week	607	233 (38.4)	1.0	
	>1 week	93	28 (30.1)	1.4 [0.9 - 2.3]	1.4 [0.8 - 2.5]
Reminder visit by CHW	No	153	78 (51.0)	1.0	
	Yes	533	353 (66.2)	1.9 [1.3 - 2.7]	1.7 [1.2 - 2.7]

The other factors assessed but were found not to be significantly associated with compliance with referral advice included; marital status and educational status of caretaker, age of head of household, social economic status of household, sex of newborn and his/her birth order at referral, place of delivery, number of times CHW visited caretaker before and after delivery, and symptoms exhibited by the newborn at time of referral, including; difficulty in breathing, fever, feeling cold, yellowing of body, severe skin rash, vomiting , excessive crying , dirty cord, diarrhea, low birth weight, and difficulty in breastfeeding. These are not included in the Table 4.

## Discussion

This study is one of the few that report newborn referral compliance in SSA. We report compliance within 24-hours, with community to health facility referral by CHWs for both sick and healthy newborns unlike other authors who focused on only sick newborns [17,24,25,26]. The factors (age of mother, health status of newborn at referral and a reminder visit by a CHW) that were significantly associated with newborn compliance differed from those documented in studies done in south Asia. The findings of this study support the evidence of the positive role CHWs can play in enhancing newborn care seeking in rural Uganda and other similar settings. 

### Compliance with newborn referral and role of community health workers

We found a relatively high level of timely newborn referral compliance for both sick (74%) and healthy newborns referred for immunisation and postnatal care (53%), respectively. Referral of healthy newborns for immunisation and postnatal care was of interest because many of them are born outside facilities in this setting [4]. Attending postnatal care gives a chance to identify and treat newborns that may exhibit a danger sign including congenital abnormalities that may have been missed by CHWs. 

Ninety-one percent of the caretakers completed referral regardless of the time taken to seek for referral care, similar to the completion rate of 87-95% reported by Nsibande et al., in South Africa [25] and higher than 86% compliance rate reported by Kirkwood et al., in Ghana [26]. Our study found a higher compliance rate of 74% among sick newborns compared to that found by Darmstadt et al., of 54% and by Baqui et al., of 32% among similar newborns referred by CHWs in southern Asia [17,24]. 

The higher level of compliance we found could have been due to the health system strengthening and regular meetings between CHWs and health workers conducted during the UNEST intervention which probably increased the communities’ trust in health facilities. The health system strengthening was done through training and reinforcing health workers’ knowledge and skills in newborn care and providing some resuscitation equipment like ambubags and masks, nasal gastric tubes, drugs such as gentamycin and injection ampicillin to treat newborn infections. 

Further, the higher level of compliance with CHW referrals in our study could also be attributed to the several interactions made between the mothers and the CHWs before and after delivery as part of the UNEST intervention activities [19]. During antenatal visits, mothers are encouraged to deliver in health facilities and to immediately seek care from health facilities whenever they recognise maternal and newborn dangers signs. The same messages were re-echoed during the postnatal home visits made by the CHWs. Further, over 95% of pregnant mothers in this setting attend at least one facility based antenatal visit before delivery [4], during which similar messages are emphasized. All this could have contributed to promote compliance with community newborn referrals. These findings support existing evidence regarding the significant role CHWs can play in promoting compliance with newborn referrals [11,26,27]. 

### Factors associated with newborn referral compliance

Older mothers (25-34 years) were less likely to comply with newborn referral possibly because they are more familiar and have experience with newborns compared to young mothers who are likely to be first time mothers. Together with the increasing responsibilities as one gets more children, these factors could prevent the older mothers from complying with newborn referrals. Whereas we found age of the mother to be associated with compliance with referral, Darmstadt et al., 2010 did not do so [17]. 

We also found that health status of the newborn at the time of referral was a significant determinant of compliance by the caretakers. Although we did not assess severity of symptoms of sickness, it is possible that caretakers responded depending on the perceived severity of symptoms of the sickness, such that caretakers of newborns referred for immunization and postnatal care were less motivated to comply with referral advice since the babies were presumably health. Previous studies have also found severity of symptoms of sickness to be associated with newborn referral compliance [12,17]. Similarly, health status of a child was associated with compliance with referral from community to health facilities among children aged below five. Those categorised as ‘urgent for referral’ were more likely to access referral care than those in the non urgent category (p=0.016) [15]. 

A reminder visit by CHWs to referred newborns within 24 hours after counselling the caretaker to seek referral care was significantly associated with timely compliance. A follow-up visit to a sick newborn was one of the activities emphasized to CHWs during their training. The reminder visit possibly achieved two purposes; first, as a reminder to the caretaker to seek referral care for the newborn, and secondly as an emphasis to the caretaker regarding the importance of seeking referral care for the newborn survival. This finding reflects the importance and contribution of CHWs in the implementation of newborn survival strategies in this setting. It also adds to the existing body of evidence that CHWs can play an important role in the promotion of newborn health [7,13,17,28,29,30]. Therefore, there is need to emphasise reminder visits to sick newborns in the CHW training guidelines. 

Darmstadt et al., found that mothers of newborns aged 0-6 days were 30% less likely to comply with referral than those of older newborns [17].However, in our study, age of the newborn, was not found to be significantly associated with newborn referral compliance. Factors that have been documented to be associated with referral compliance among older children include; severity of disease [12,17], functionality of the health system [31], knowledge and perceptions of caretakers about danger-signs in the sick child [32,33] and provision of a referral slip by health workers [34]. 

## Methodological Consideration

The strength of our work was that we attempted to trace and study all healthy and sick newborns referred by CHWs unlike previous studies that assessed only sick newborns [17,25,28]. However, our study also had limitations; first, we attempted to verify if caretakers reached the health facilities after receiving referral advice but due to the poor record keeping in health facilities in this setting we were unable to achieve this task. We instead used self report similar to other studies [12,14]. 

Secondly, we conducted the assessment after one and half years after the initial start of the intervention. This could have created recall bias making some information less accurate than it would have been, had it been collected immediately after the CHWs gave referral advice to the caretakers. In the effort to minimise recall bias we directly picked some of the information from the referral forms. This data consisted of newborn age at referral, date of referral and reason for referral. 

Also to reduce the response bias that could have occurred, we used clear, precise and short questions and avoided leading ones. We also trained the data collectors to create rapport with the respondents and enable them answer the questions freely.

Thirdly, our study included the 700 caretakers of newborns that were referred. The question that arises is whether our study had enough power to detect the factors associated with timely compliance to newborn referral. We assessed the power of this study, assuming that the most important independent variable was the newborn being sick or not, and used different values of the prevalence of sick newborns among caretakers with timely compliance and non sick children. We used the Power and Sample size software [35] to compute the power of our study. Table 5 shows a summary of our assessment, and it is clear from this table 5 that this study had a power of more than 80% to detect an odds ratio of at least 1.6, if the prevalence of sick newborns among caretakers with timely compliance was at least 33%. The prevalence of sick newborns among caretakers with timely compliance in our study was 55%.

**Table 5:** Power analysis for this study using a sample size of 700.

**Proportion (%) of the sick newborns among the timely compliers**	**Odds ratio (OR)**	**Power**
**0.55**	2.0	>99
**0.50**	2.0	>99
**0.45**	2.0	>99
**0.40**	2.0	>99
**0.35**	2.0	>99
**0.30**	2.0	>99
**0.30**	1.9	98
**0.30**	1.8	96
**0.30**	1.7	91
**0.30**	1.6	82
**0.30**	1.5	69

Fourthly, this was a trial setting, where the CHWs received focused training and supervision on newborn care, so our findings may not be generalised to the general health system. We also did not assess the quality of care at the referral centres, as this was outside the scope of this study, but will be reported in a subsequent study.

Lastly, the compliance level we found could have been affected by way of measurement when we defined compliance as positively responding within 24-hours to seek referral care. A shorter response period after referral advice could have been considered given that newborns exhibiting danger signs require immediate care otherwise; 24-hour delay may result into death. However, given that multiple factors including individual, household and health facility interplay to complete referral, a 24-hour period was deemed most realistic and has been used previously by other authors to define delay in compliance with referral advice [15,17]. Further research is recommend using qualitative methods to explore and understand social cultural factors that may influence newborn referral compliance in this setting.

### Policy Implications

In Uganda, newborn care has been introduced in the integrated community case management program under which community health workers (referred to as Village Health Teams (VHTs) identify and refer sick newborns [29] As this program is rolled out in the country, there are lessons to learn from our findings including; i) CHWs can effectively make a link between community and health facility, through initiating referrals for sick and healthy newborns and ii) caretakers of the newborns can pay attention to CHWs’ counseling and comply with the referral advice particularly if the CHWs are encouraged to make an extra visit to the caretakers after recommending newborn referral care. These lessons add to the previous evidence generated, showing that trained and effectively supervised CHWs can identify and refer sick newborns in a rural setting [36]. We are also in the process of generating more evidence on the health facility readiness to manage sick newborns in the same setting.

## Conclusion

Compliance of caretakers with community-to-facility newborn referrals initiated by CHWs was high in this setting. Acute illness, being born to a younger mother and a reminder visit by the CHW to a referred newborn were predictors of compliance with newborn referral in this study. In similar contexts integration of CHWs into maternal and newborn care programs has the potential to increase health facility based care seeking for newborns that may contribute to reduction of newborn mortality.
